# Effectiveness of an intrauterine device informative intervention among post-natal women in Western Jamaica

**DOI:** 10.1186/s12978-021-01075-1

**Published:** 2021-01-21

**Authors:** Sarah G. Franklin, Maya O’Neal, Ambreanna Arneus, Calvin Colvin, Maung Aung, Pauline E. Jolly

**Affiliations:** 1 Department of Epidemiology, School of Public Health, University of Alabama at Birmingham, 1665 University Blvd, Birmingham, AL 35233 USA; 2 Epidemiology and Research Unit, Western Region Health Authority, Ministry of Health and Wellness, Lot 31B, Fairview Shopping Centre, Montego Bay, Jamaica

**Keywords:** Contraceptive use, Intrauterine device, Reproductive health

## Abstract

**Introduction:**

Intrauterine devices are the most effective long-acting reversible contraceptives, but in many developing countries, such as Jamaica, these devices remain underutilized.

**Methods:**

A cross-sectional informative intervention was conducted among women ≥ 18 years of age attending postnatal clinics in western Jamaica from May to August 2018. Data were collected using an investigator-administered questionnaire/pre-test followed by a 12-slide PowerPoint® presentation and a post-test.

**Results:**

Most of the 299 women who participated were 18–29 years of age, with a mean age of 27.1 (SD ± 6.1) years. Most had their first pregnancy between ages 18 and 24 years, with mean age at first pregnancy of 20.2 (SD ± 4.0) years. Only 3.0% of participants reported current use of an intrauterine device; 3.5% reported using an intrauterine device in the past. For nearly every measure of knowledge of intrauterine devices, there was a significant change in the proportion of participants who got the correct answer from the pre-test to the post-test. The mean summed pre-test knowledge score was 9.54 (SD ± 3.46) and the post-test score was 15.23 (SD ± 1.92); the possible total score is 18. The difference between the mean scores (5.69 points) was also significant.

**Conclusion:**

The intervention resulted in significant change in knowledge of intrauterine devices among the women and cleared up many misconceptions that may have contributed to reluctance of women to use intrauterine devices. Women of reproductive age in Jamaica should be counseled on contraceptive methods including intrauterine devices so that these devices can be considered in their contraceptive choices.

## Introduction

IUDs have increased worldwide in popularity as a method of long-acting reversible contraceptives (LARC) [1]. Yet, in several developing countries such as in Latin America and the Caribbean the rate of use of LARCs (including IUDs) is < 10% [2]. According to a report published by the United Nations, 71.8% of married/in-union women aged 15–49 years in Jamaica in 2015 reported using contraceptives, but only 1.0% of these women reported using an IUD [3]. This percentage was the second lowest after Haiti when compared to other Latin American or Caribbean countries reported in this study; 37.8% of married/in-union women in Haiti reported using any contraceptive method and only 0.1% reported IUD use [3].

Modern IUDs are the most effective form of LARCs; the known side effects include mild pain for a few days after IUD insertion, irregular menstrual cycles, spotting between periods, or heavier menstrual flow [4, 5]. The low failure rate of IUDs in preventing pregnancy provides them an advantage over other forms of contraception, especially in countries where abortions are unsafe and/or illegal [5, 6]. According to a report by the World Health Organization, an IUD “offers, on average, three times the length of contraceptive protection offered by other modern reversible methods”; thereby providing women greater control over their reproductive choices [1, 3, 7–10].

Addressing and encouraging the use of IUDs among women in the Caribbean may prove advantageous as the most widely used contraceptive methods among married/in-union women in this region in 2015 were female sterilization (21.9%), birth control pills (9.9%), male condoms (9.6%), and injectable contraceptives (8.5%) [3]. Since the pill can be ineffective if the woman does not adhere to the daily schedule, some women may see sterilization as the only long-term method of contraception available [1]. Therefore, women could find IUDs appealing if they wish to space out their pregnancies.

Of women who used contraceptives in Jamaica, the most widely used methods were male condoms (25.1%), the birth control pill (17.2%), and injectable contraceptives (13.7%) [3]. According to reports published by the Jamaican National Family Planning Board in 2000, knowledge about contraceptive use and sex are high among Jamaican adolescents and young adults; 85% of females and approximately 72% of males ages 15–19 years stated that they received sexual education information in or out of school [11]. Yet, the rate of contraceptive use among sexually active teenagers was only 69%. In this same report, 40% of women < 20 years of age reported being pregnant at least once, and 85% of those were unintended pregnancies [11, 12].

The main barrier that has been observed towards IUDs and other LARCs in Jamaica is access to, and/or attitudes towards, this method of contraception. Since some previous studies have found that knowledge about IUDs are not a major barrier among Jamaican women, access to clinics which provide contraception could be the main barrier to the use of IUDs and other LARCs [11, 12]. It has also been observed by studies conducted in other countries that a rather important barrier to IUD use is a lack of healthcare providers (HCPs) who are trained in how to perform an IUD insertion [2, 13–15]. Knowledge and attitudes about IUDs among the sexually active female population in Jamaica could also be a factor. In the more rural areas of Jamaica, some women may only know about contraceptive methods that are more commonly used such as the male condoms, contraceptive pills, injection, or sterilization [2, 12, 16]. The primary objective of this research was to conduct an informative intervention on IUDs among women attending postnatal clinics in western Jamaica. Secondary objectives were to determine the prevalence of IUD use and identify factors contributing to low use.

## Methods

### Study design and participant recruitment

A cross-sectional informative intervention study was conducted among 299 women ≥ 18 years of age attending selected postnatal care clinics in the four parishes of western Jamaica (St. James, Westmoreland, Hanover and Trelawny) under the Western Regional Health Authority (WRHA). A convenience sampling method was used. Regional public health nurses informed the women of the study when they visited the clinic for their appointments and asked if they would be interested in participating. Those who expressed interest in participating were introduced to the research staff for further information on the study and to have their questions answered. Informed consent was obtained from each woman who volunteered to participate. The response rate was 95%.

### Sample size calculation

Based on the United Nations report in 2015 that only 1.0% of married/in-union Jamaican women of reproductive age (15–49 years) reported using an IUD, the sample size for the current study was determined to be a minimum of 16 women with a 95% confidence limit and 5% margin of error [4]. The sample was calculated using the online calculator EpiTools by Ausvet© (http://epitools.ausvet.com.au). Our sample of 299 women was more than adequate for the study given the level of precision.

### Data collection

Participant data were collected using an investigator-administered questionnaire/pre-test followed by an informative intervention and a post-test. The questionnaire/pre-test was divided into seven sections, including sociodemographic information (age, education level, religious affiliation, marital status, occupation, and income), health seeking behaviors, family planning information, contraceptive questions, IUD use, knowledge of IUDs, and sexual risk behaviors.

After administration of the questionnaire/pre-test, a short 12-slide PowerPoint® presentation which lasted approximately 20 min was made by the study staff. The presentation provided general descriptive information on IUDs such as how they are shaped and how they prevent pregnancy using copper wire or by releasing hormones into the body and how they are inserted and removed from the uterus. The presentation also addressed the effectiveness of IUDs compared to other forms of contraception, common myths about IUDs, and the benefits and possible side effects. During and after the presentation, participants were encouraged to ask any questions they had regarding IUDs and contraceptive use. Following the presentation, a one-page post-test that included the same questions from the pre-test was administered to re-assess the participants’ gain in IUD knowledge.

With regards to confidentiality, the women were informed that the information collected from their questionnaires/tests would be kept strictly confidential and that the compiled study results would be shared with health officials at the WRHA and published for scientific purpose without revealing their identity. Administration of the questionnaire/tests and PowerPoint presentation was conducted in private clinic rooms. Unique ID numbers were used instead of names and were placed on the questionnaires. Only the research staff had access to the questionnaires which were stored in locked cabinets. The study dataset was kept on a password protected computer with encrypted hard-drive.

### Statistical analyses

Appropriate descriptive measures were used to describe sociodemographic characteristics and reproductive health behaviors of the study group. McNemar’s tests were used to determine whether there were any significant differences in pre- and post-test answers for each individual knowledge question. The number of correct answers on the pre and post-tests were then summed to generate a cumulative knowledge score. A paired t-test was used to assess the change in mean cumulative knowledge score between the pre- and post-test and alpha of 0.05 was used to determine statistical significance. All analyses were conducted using Statistical Analysis System (SAS; Cary, North Carolina, USA) software version 9.4.

## Results

Table 1 describes the sociodemographic characteristics of the study population. The majority of the women were 18–29 years of age, with a mean age of 27.1 (SD ± 6.1) years. Over half (53.85%) earned less than the Jamaican minimum wage of approximately J$28,000 a month for full-time employees at the time of the study. Participants’ reproductive history and use of contraceptives are described in Table 2. A majority of the women had their first pregnancy between ages 18 and 24 years, with a mean age of 20.2 (SD ± 4.0) years. Most women had 1 or 2 children. Only 9 women reported current IUD use (3.0%), and 10 reported previous IUD use (3.5%). Over half (52.5%) reported having been told unpleasant things about IUDs.

**Table 1:** Sociodemographic characteristics of the study population (N = 299)

Characteristics	N (%)
Parish	
Hanover	26 (8.7)
St. James	169 (56.5)
Westmoreland	46 (15.4)
Trelawny	58 (19.4)
Age (years)	
18–24	117 (39.1)
25–29	86 (28.8)
30–34	52 (17.4)
35–40	39 (13.0)
> 40	5 (1.7)
Highest education level	
< Secondary	64 (21.4)
Secondary	175 (58.5)
Technical/Vocational/College/University	60 (20.1)
Marital status	
Single	149 (49.8)
Married	34 (11.4)
Common Law	108 (36.1)
Widowed/divorced/other^a^	8 (2.7)
Current occupation	
Unemployed	116 (38.8)
Unskilled	78 (26.1)
Clerical	22 (7.4)
Skilled	26 (8.7)
Professional	27 (9.0)
Other^b^	30 (10.0)
Individual monthly income	
< J$24,800	161 (53.8)
J$24,800–60,000	75 (25.1)
J$61,000–120,000	42 (14.1)
> J$120,000	17 (5.7)
Other^c^	4 (1.3)

^a^Other includes: engaged and separated

^b^Other includes: student, self-employed, and entrepreneur

^c^Other includes: refused and does not know

**Table 2:** Reproductive history and contraceptive use by the study population (N = 299)

Variables	N (%)
Age at first pregnancy (years)	20.2 (± 4.0)
Number of pregnancies	
1	92 (30.8)
2	87 (29.1)
3	59 (19.7)
≥ 4	61 (20.4)
Number of children	
0	7 (2.3)
1	117 (39.1)
2	88 (29.4)
3	45 (15.1)
≥ 4	42 (14.1)
Unplanned pregnancies	
None	96 (32.1)
Some	97 (32.4)
All	106 (35.5)
Currently using contraceptives	
No	231 (77.3)
Yes	68 (22.7)
Method of contraception currently used	
Male condoms	32 (10.7)
Hormonal injection	18 (6.0)
Birth control pills	12 (4.0)
Sterilization	5 (1.7)
Discussed contraception with a health provider	
No	97 (34.1)
Yes	187 (65.9)
Received information about IUDs from health provider	
No	55 (29.4)
Yes	132 (70.6)
Currently using an IUD	
No	290 (97.0)
Yes	9 (3.0)
Previously used an IUD	
No	279 (96.5)
Yes	10 (3.5)
Has been told unpleasant things about IUDs	
No	142 (47.5)
Yes	157 (52.5)

The results of the pre- and post-test analysis (Table 3) show that for nearly every measure of knowledge, there was a significant change in the proportion of participants who got the correct answer from the pre-test to the post-test. Results of note include that there was a 41.8 percentage point increase in participants’ belief that IUDs were safe and had a low risk of side effects, a 43.8 percentage point decrease in participants’ belief that IUDs could puncture one’s womb during insertion, and a 38.8 percentage point decrease in participants’ belief that IUDs could cause infertility. There was a 40.1 percentage point decrease between the pre- and post-test results on participants’ belief that IUDs were difficult to remove. The mean summed pre-test knowledge score was 9.54 (SD ± 3.46) and the post-test score was 15.23 (SD ± 1.92); the possible total score was 18. The difference between the mean scores (5.7 points) was also significant. Two non-significant measures of knowledge of IUDs, namely IUDs can increase risk of anemia and IUDs do not protect against sexually transmitted diseases or HIV, were not covered in the educational intervention. These omissions should definitely be included when the intervention is conducted in the future.

**Table 3:** Numbers and percentages of women who obtained correct answers to pre-test/post-test questions and mean differences in test scores (N = 299)

Assessment questions	Pre-test N (%)	Post-test N (%)	% or mean difference	P-value^a^
IUDs are a reversible method of contraception	219 (73.2)	289 (96.7)	23.42	** < 0.001**
IUDs are extremely difficult to remove	145 (48.5)	25 (8.4)	40.1	** < 0.001**
A woman can have mild cramps for a few days after IUD insertion	179 (59.9)	289 (96.7)	36.8	** < 0.001**
IUDs increase the chance of pregnancy in the tubes	137 (45.8)	60 (20.1)	25.8	** < 0.001**
There are 3 forms of IUDs	263 (88.0)	105 (35.1)	52.8	** < 0.001**
A woman can become pregnant as soon as the IUD is removed	209 (69.9)	286 (95.7)	25.8	** < 0.001**
IUDs can increase risk of anemia	58 (19.4)	70 (23.4)	4.0	0.140
IUDs can cause a woman to become infertile after it is removed	190 (63.6)	74 (24.8)	38.8	** < 0.001**
IUDs often puncture the womb during insertion	177 (59.2)	46 (15.4)	43.8	** < 0.001**
IUDs do not protect against sexually-transmitted diseases or HIV	241 (80.6)	246 (82.3)	1.7	0.564
IUDs can be easily removed by a doctor pulling on the string	172 (57.5)	288 (96.3)	38.8	** < 0.001**
IUDs can cause longer and heavier menstrual flow	153 (51.2)	261 (87.3)	36.1	** < 0.001**
IUDs are the most effective method of birth control	153 (51.2)	270 (90.3)	39.1	** < 0.001**
IUDs expire and are no longer effective after a number of years	198 (66.2)	256 (85.6)	19.4	** < 0.001**
IUDs keep sperm from reaching/fertilizing the egg	260 (87.0)	297 (99.3)	12.4	** < 0.001**
IUDs are easy to insert with one doctor visit	221 (73.9)	292 (97.7)	23.8	** < 0.001**
IUDs cause infections	241 (80.6)	47 (15.7)	64.9	** < 0.001**
IUDs are very safe and risk of side effects is very low	149 (49.8)	274 (91.6)	41.8	** < 0.001**
Mean (SD) of Cumulative IUD Knowledge Score	9.54	15.23	5.69	** < 0.001**

Bold = significant at p < 0.05

^a^P-values generated from McNemar’s tests or Paired T-Test, as appropriate

## Discussion

This was the first informative health intervention specifically on IUD use and knowledge among postnatal women in Jamaica. While most women possessed general knowledge about IUDs, several of the participants incorrectly answered questions regarding misconceptions about IUDs on the pre-test. Some of the misconceptions were that IUDs are extremely difficult to remove, increase risk of ectopic pregnancy, can cause infertility, and cause infections. This finding is similar to other studies that found common misconceptions about IUDs; including misconceptions that IUDs can puncture the womb, cause infertility, and be used to induce abortions.[2, 3, 6, 17–20]

Although the majority of women who reported discussing contraception with their HCP said that they also received information about IUDs (70.6%), many responded incorrectly to the IUD knowledge questions on the pre-test, showing insufficient communication or understanding regarding IUDs. Previous studies have found that HCPs in developing countries often do not possess the knowledge about IUDs that is needed to appropriately inform their patients, and that HCPs’ beliefs and attitudes towards IUDs and other forms of contraceptives can affect the information that they provide to their clents [8, 13, 15].

Similar to studies conducted in other countries, the findings of this study suggest that an informative intervention can be effective in improving knowledge and attitudes regarding contraceptives and IUDs [7, 9, 13, 20, 21]. The intervention used in this study was similar to those used in studies that we previously conducted on increasing knowledge on cervical, breast, and prostate cancers in western Jamaica and was successful at dispelling common misconceptions about IUDs and their safety [22–25]. We observed significant decreases in participants’ beliefs that IUDs can cause ectopic pregnancy (from 45.8% on the pre-test to 20.1% on the post-test), cause infertility (from 63.6 to 24.8%), and puncture the womb during insertion (from 59.2 to 15.4%). We also observed significant increases in participants’ beliefs that IUDs are the most effective form of contraception without sterilization (from 51.2 to 90.3%), are safe with a low risk of side effects (from 48.9 to 91.6%), and can be easily removed (from 57.5 to 96.3%).

In Jamaica, the cost of IUDs has not been shown to be a barrier to access of IUDs nor to affect a woman’s willingness to receive an IUD. This is due to the implementation of the abolition of user fees policy in 2008 by the Government of Jamaica. Under this policy, no Jamaican pays a fee for health services, including IUDs, at public health facilities [26]. However, only the copper IUD is provided through the National Family Planning Board.

## Limitations

This study has certain limitations that should be considered in interpreting the results. Convenience sampling was used, and the study was limited to women attending postnatal clinics in the western region. Thus, the sample was not representative of the general population. Additionally, self-reported data might have been biased due to social desirability. The cross-sectional design of the study did not allow for long-term follow-up with participants to test long-term recall of knowledge gained during the intervention or the effect of the intervention on uptake of IUD.

## Conclusion

Regardless of the limitations, this study shows that the informative intervention resulted in significant change in knowledge for nearly all questions included in the pre- and post-test. The intervention did not only provide accurate knowledge on the safety, effectiveness, and ease of insertion and removal of IUDs, but also cleared up many myths that may have possibly contributed to reluctance of women to use IUDs. This intervention should be replicated among women of reproductive age in Jamaica to determine its effectiveness in increasing accurate knowledge and uptake of IUDs. Counseling women on contraceptive methods including IUDs will allow them to consider these devices in their contraceptive choices.

## Data Availability

The datasets used and analyzed for this are available from the corresponding author upon reasonable request.

## Abbreviations

IUD: Intrauterine device
LARC: Long-acting reversible contraceptive
HCP: Healthcare provider
WRHA: Western Regional Health Authority
